# Supplementary material for: The interactome of intact mitochondria by cross-linking mass spectrometry provides evidence for coexisting respiratory supercomplexes
Source: Mol Cell Proteomics. 2017 Dec 8;17(2):216–32. doi: 10.1074/mcp.RA117.000470 (PMC5795388; doi:10.1074/mcp.RA117.000470)
Supplement: Supplemental Data [file supp_RA117.000470_133922_0_supp_23978_fzffwf.zip › spectra_annotation/mito_spectra_annotation/270-1-7-1-6-1.pdf]

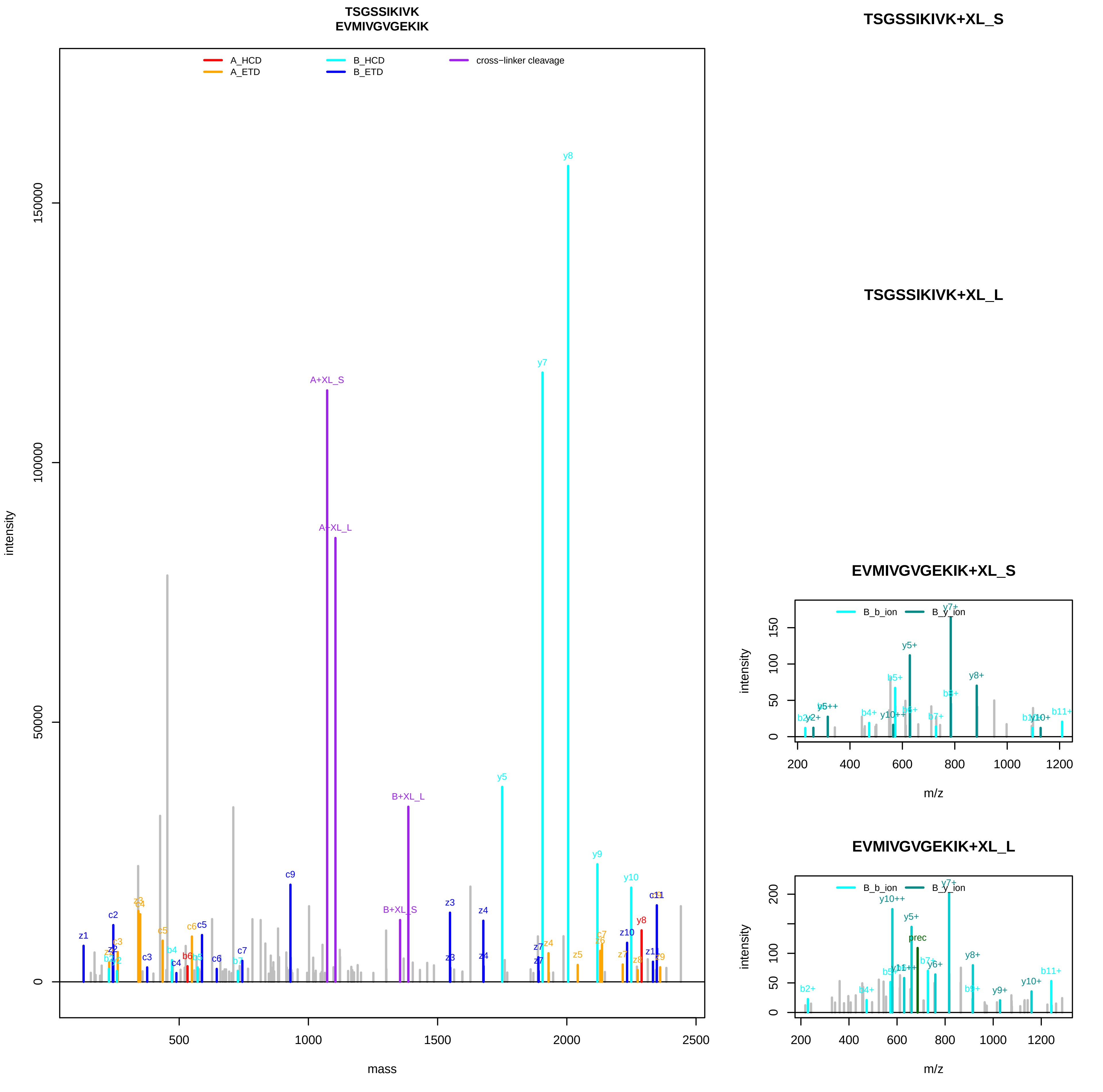
TSGSSIKIVK
EVMIVGVGEKIK
A_HCD
A_ETD
B_HCD
B_ETD
cross−linker cleavage
A+XL_S
A+XL_L
B+XL_S
B+XL_L
y8
y7
y5
y9
y10
c9
z3
z4
c11
y8
intensity
mass
0
50000
100000
150000
500
1000
1500
2000
2500
TSGSSIKIVK+XL_S
TSGSSIKIVK+XL_L
EVMIVGVGEKIK+XL_S
B_b_ion
B_y_ion
y7+
y5+
y8+
b5+
y5++
y10++
b11+
intensity
m/z
0
50
100
150
200
400
600
800
1000
1200
EVMIVGVGEKIK+XL_L
B_b_ion
B_y_ion
y7+
y10++
y5+
prec
y8+
y6+
y10+
b11+
y9+
b2+
intensity
m/z
0
50
100
200
200
400
600
800
1000
1200
